# Dengue Fever in an Adult Presenting as Immune Thrombocytopenic Purpura (ITP): A Case Report

**DOI:** 10.7759/cureus.75124

**Published:** 2024-12-04

**Authors:** Debananda Sahoo, Anupam Dey, Tuhin S Bandyopadhyay, Sujata Devi, Sunita Dalei

**Affiliations:** 1 General Medicine, All India Institute of Medical Sciences, Bhubaneswar, Bhubaneswar, IND

**Keywords:** dengue fever, haematocrit, immune thrombocytopenic purpura, platelet, thrombocytopenia

## Abstract

Immune thrombocytopenic purpura (ITP) is an autoimmune self-limiting disorder characterized by a decreased platelet count. Usually, it affects children after viral infections. Adults often develop chronic ITP, but they can also develop ITP following viral infections, which is uncommon. A decrease in platelet synthesis from megakaryocytes and a reduction in platelet half-life appear to cause post-viral thrombocytopenia. Most clinical signs of post-viral thrombocytopenia appear towards the end of the first week of illness, but if they appear after the second week of illness, ITP development should be considered. Although thrombocytopenia is frequently associated with dengue fever, reports of ITP as a presenting symptom are less common. We describe a female patient in her forties who presented with ITP as the initial symptom of dengue fever. The patient was successfully managed with supportive care and platelet transfusions. This case highlights the importance of considering ITP as a potential complication of dengue fever and emphasizes the need for early diagnosis and appropriate management.

## Introduction

Immune thrombocytopenic purpura (ITP), also known as idiopathic thrombocytopenic purpura or Werlhof's disease, is a diagnosis of exclusion that stands out due to its characteristic thrombocytopenia. ITP is commonly categorized based on its duration since diagnosis (recently diagnosed, persistent, chronic, and refractory ITP) or its etiology (primary versus secondary) [1]. While secondary ITP accounts for 20% of diagnoses, primary ITP in adults has an annual incidence of 3.3 per 100,000 people, with a prevalence of 9.5 per 100,000 adults [2]. The formation of antibodies against platelets leading to reduced half-life and potential inhibition of platelet release from megakaryocytes induce this phenomenon [3]. Clinically, we observe a normal peripheral blood smear, a very low platelet count, and mucocutaneous hemorrhage, purpura, or ecchymoses.

The clinical diagnosis of ITP was made after ruling out alternative options. Drugs (aspirin, digoxin, phenytoin, etc.), cancers (lymphoma and adenocarcinoma), common variable immunodeficiency, autoimmune disorders (thyroid disease, autoimmune hepatitis, systemic lupus erythematosus), and infections (human immunodeficiency virus, hepatitis C, varicella-zoster virus, rubella, etc.) can all induce ITP [4-5]. Adults between the ages of 20 and 50 years typically manifest chronic ITP, which usually does not precede viral infections and has a 3:1 woman-to-man preponderance [6]. In contrast, among children, ITP emerges following a viral infection and is generally self-limiting, with recovery occurring within three months in most of the cases. Viral infection appears to cause thrombocytopenia through a decrease in platelet synthesis from megakaryocytes and a decrease in platelet half-life. The latter is the primary mechanism [6]. Reticuloendothelial cells, particularly in the spleen, destroy platelets sensitized by autoantibodies [7, 8]. Eighty percent of individuals exhibit autoantibodies against platelet membrane glycoproteins [9, 10].

Dengue fever, commonly referred to as break bone fever, seven-day fever, or dandy fever, is one of the most common mosquito-borne viral illnesses spread by arthropods worldwide. Globally, there are around 100 million new cases of dengue each year, and 2.5 billion people reside in dengue-risk areas [11]. Low white blood cells, low platelets, and an increased haematocrit level in the blood are the hallmarks of dengue fever. Fever, headache, muscle aches, maculopapular rash, pain behind the eyes, and bleeding are among the symptoms [12]. Dengue fever causes thrombocytopenia, which has an incidence of 40.3% [13]. Antiplatelet antibodies, disseminated intravascular coagulation (DIC), early bone marrow suppression, and peripheral sequestration of platelets are believed to cause it. The condition is most common during the critical phase of the illness (three to four days after the onset of fever), which lasts for 36-48 hours and then gradually improves during the convalescent phase. There are very few case reports that show both a temporal and a causal relationship between immune thrombocytopenic purpura development and dengue fever, mostly involving youngsters [14]. There is no definitive agreement or publicly available statistics regarding the prevalence of ITP following dengue virus infection. This case report highlights a woman in her forties who was admitted to a tertiary care facility in eastern India due to dengue fever, which manifested as ITP.

## Case presentation

A young woman in her forties recently received a type 2 diabetes diagnosis without any prior medication intake history. A few weeks ago, she suffered from a high temperature, chills, and rigor, as well as myalgia and back pain, which she managed with medication. She had been experiencing repeated episodes of melena, gingival bleeding, and hematuria for the previous five to six days when she arrived at the emergency department. She had also experienced clear, non-projectile vomiting, including food particles, for the previous three days. There was no previous medical history of weight loss, recurring infections, burning micturition, joint discomfort, oral ulcers, alopecia, heat or cold intolerance, or drug use. She denied any recent travel. Previously, reports from a nearby hospital consistently indicated that the patient had received eight units of random donor platelet (RDP) transfusion and had a platelet count of less than 10,000/cumm. Upon examination, we found the patient alert, afebrile, and cognizant of time, place, and people. We measured her blood pressure in the right arm while supine, measuring 120/80 mmHg; pulse rate was 74 bpm; respiratory rate was 18/min; and oxygen saturation (SpO_2_) was 98% at room air. We found no organomegaly and other systemic examination results were within normal ranges.

Investigations and outcomes

The complete blood count (CBC) showed total platelet count (TPC) to be less than 5000/cumm on the first day of admission, which corresponds to the 15-16th day after the fever started. The Dengue IgM test yielded positive results, and the TPC was consistently less than 10000/cumm in the serial CBC measurements. Throughout her hospital stay, the patient got three random donor platelet (RDP) units and one single donor platelet (SDP) unit. Urine routine and microscopy (R/M) and culture and sensitivity (C/S) were both negative. The results of various laboratory investigations are shown in Table 1, indicating all are normal except TPC and hematocrit (PCV).

**Table 1:** Laboratory Results TLC: Total leucocyte count, Hb: hemoglobin, TPC: total platelet count, PCV: packed cell volume, INR: international normalized Ratio, MP ICT: malaria parasite immuno-chromatographic test, ANA IFA: anti-nuclear antibody by immuno-fluorescence assay, C3: complement 3, C4: complement 4

Investigations	Results	Reference range
TLC	3,900/dl	4,000-11,000/dl
Hb	14.6 gm%	12-15 gm%
TPC	5000/dl	1.5 to 4 lakh/dl
PCV	48%	40(±6)%
HbA1C	5.9%	4.0-5.6%
FBS	97 mg%	75-100 mg%
INR	0.93	0.8-1.2
Serum C3	94 mg%	83-177 mg%
Serum C4	37 mg%	16-47 mg%
MP ICT	Negative	
ANA IFA	Negative	
ANA Profile	Negative	

Ultrasound of the abdomen and pelvis and X-ray chest PA view were done as a part of routine investigations and revealed no specific abnormality. We made a provisional diagnosis of ITP (post-dengue infection) due to thrombocytopenia that persisted into the third week of dengue fever, mandating transfusion. A bone marrow test revealed no additional abnormalities, as well as a higher number of normal and slightly dyspoietic megakaryocytes. These results are consistent with ITP, which is likely followed by dengue. Adults with immunological thrombocytopenic purpura often require treatment with oral prednisone at the outset (1-1.5 mg/kg/day) [15]. Most individuals with ITP begin to respond to prednisone after two weeks of treatment [16, 17]. In anticipation of secondary ITP, we started the steroid treatment as per the recommendation. The platelet counts gradually rose, beginning on the fourth day of hospitalization (perhaps the 19th or 20th day after the fever started). The patient showed progressive recovery with additional supportive therapy like intravenous fluids, proton pump inhibitor (PPI), antipyretics, and other measures; at the time of discharge (on day 23rd or 24th day), the platelet count was 129,000/cumm.

Follow-up

Upon assessment two weeks later in the Medicine outpatient department (OPD), the patient's liver and kidney parameters as well as her platelet count (TPC 255,000/cumm), were all normal, and there had been no observable bleeding symptoms. The patient is currently improving, continues to receive routine follow-up, and intends to taper the steroid after four weeks of treatment.

## Discussion

When thrombocytopenia persists after the second week of dengue infection, it should raise suspicions about other possible causes and prompt medical attention. Once we rule out other potential causes of thrombocytopenia, such as drug usage, dietary deficiencies such as vitamin B12 deficiency, toxins, autoimmune diseases such as autoimmune thyroiditis, systemic lupus erythematosus (SLE), and primary bone marrow diseases, we should consider ITP as one of the differential diagnoses. Particularly among adults, the number of case reports demonstrating a connection between dengue and ITP is quite low [14].

In the current dengue case, there was no record of platelet count before the illness. However, given that this was a primary dengue virus infection, it is probable that the attack on the platelets began with megakaryocyte malfunction and direct virus damage to the platelets [18]. Immunological processes most likely sustained low platelet counts, indicating a post-dengue ITP state. This diagnosis is supported by the fact that skin mucosal bleeding and melena did not appear until the end of the second week of the illness. This is because immunological thrombocytopenia typically does not appear until the end of the second week of illness [19], but thrombocytopenia induced by an infectious condition appears earlier, toward the end of the first week.

We do not fully understand the mechanism by which dengue fever can lead to ITP. It is believed that viral infection may trigger an autoimmune response that targets platelets [14]. Prompt identification and treatment are also necessary to prevent potentially catastrophic outcomes, such as brain hemorrhage. A high mean platelet volume (MPV) indicates that patients are experiencing more platelet breakdown. MPV is frequently high or normal in dengue patients; hence, increased platelet destruction could be the primary cause of thrombocytopenia. In adults, ITP is typically a chronic, idiopathic condition. However, as proven in the current case, ITP can manifest in a chronic form, a combination of adult and juvenile forms, following viral infection. The parameters influencing whether post-viral thrombocytopenia will follow an acute or chronic course remain unknown. It is believed that, in some immunologically predisposed individuals, the persistence of virus-induced antibodies against platelets is the cause of a chronic rather than acute course of thrombocytopenia [20]. As the primary culprit for this thrombocytopenia is viral infections (here is dengue virus), the preventive measures for the spread of the infection like the use of insect repellents, full sleeve shirts and pants, mosquito nets, and measures to control the breeding of mosquitoes in and around of our homes is of primary significance.

## Conclusions

Dengue thrombocytopenia can be caused by decreased bone marrow cell production or accelerated platelet breakdown and clearance from the peripheral circulation. Irrespective of the mechanism of thrombocytopenia, the future course of the illness is unpredictable, making it more necessary for proactive treatment. Before connecting dengue infection with ITP, it is necessary to rule out other possibilities, such as systemic lupus erythematosus and lymphoproliferative disorders. The earliest identification and treatment of the condition can mitigate the development of catastrophes. Preventive measures to control the dengue infection spread by controlling mosquito bites in society are of paramount importance. It requires further large-scale studies to establish a causal relationship between dengue and ITP.
